# Supplementary material for: Effects of Different Land Use Types and Soil Depths on Soil Mineral Elements, Soil Enzyme Activity, and Fungal Community in Karst Area of Southwest China
Source: Int J Environ Res Public Health. 2022 Mar 7;19(5):3120. doi: 10.3390/ijerph19053120 (PMC8910417; doi:10.3390/ijerph19053120)
Supplement: Supplementary file 1 [file ijerph-19-03120-s001.zip › ijerph-1602648-supplementary.pdf]

Supplementary Materials

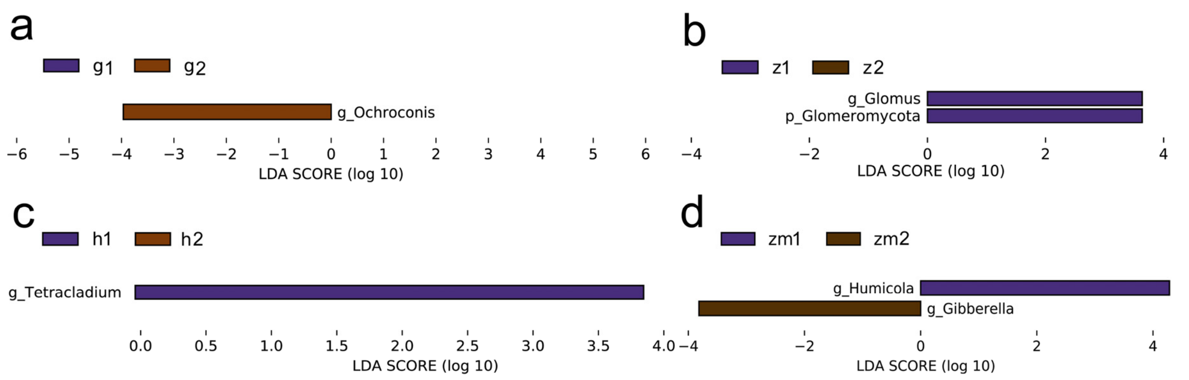

Figure S1. LDA of soil fungi with statistical difference between 0–20 cm and 20–40 cm soil depth in different land use types.

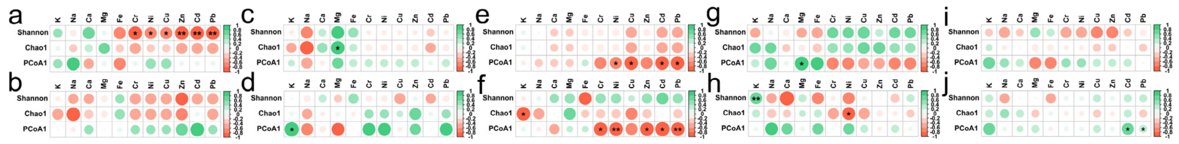

Figure S2. Spearman's Rank correlation coefficients between soil metal element content and soil fungal community variety.

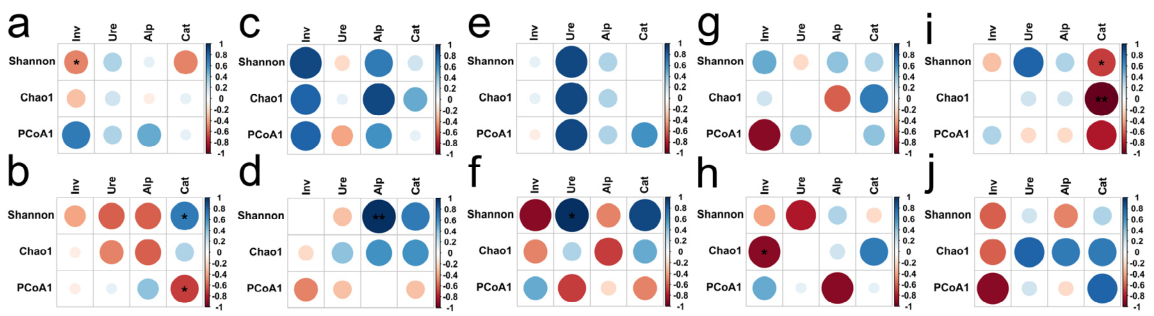

Figure S3. Spearman's Rank correlation coefficients between soil enzyme activity and soil fungal community variety

Table S1. Results of two-way ANOVA for the effects of land use types (LUT) and soil depths (SD) on soil contents of K, Na, Ca, Mg and Fe, F: F-value.

Treatments	df	K		Na		Ca		Mg		Fe	
		F-value	*p*	F-value	*p*	F-value	*p*	F-value	*p*	F-value	*p*
LUT	4	84.1	**<0.001**	3.01	**0.029**	3.86	**0.010**	25.42	**<0.001**	13.26	**<0.001**
SD	1	1.68	0.203	0.31	0.580	1.88	0.178	2.83	0.100	0.45	0.508
LUT × SD	4	1.08	0.380	0.60	0.661	4.50	**0.004**	3.44	**0.017**	1.75	0.157

Table S2. Results of two-way ANOVA for the effects of land use types (LUT) and soil depths (SD) on soil contents of Cr, Ni, Cu, Zn, Cd, and Pb, F: F-value.

Treatments	df	Cr		Ni		Cu		Zn		Cd		Pb	
		F-value	*p*	F-value	*p*	F-value	*p*	F-value	*p*	F-value	*p*	F-value	*p*
LUT	4	2.91	**0.033**	2.55	0.054	10.26	**<0.001**	3.76	**0.011**	8.43	**<0.001**	0.83	0.514
SD	1	0.22	0.645	0.04	0.843	0.00	0.987	0.06	0.804	0.15	0.698	0.46	0.501
LUT × SD	4	2.07	0.103	2.06	0.105	3.11	**0.026**	1.78	0.151	2.02	0.110	1.92	0.125

Table S3. Results of two-way ANOVA for the effects of land use types (LUT) and soil depths (SD) on activity of Inv, Ure, Alp and Cat, F: F-value.

Treatments	df	Inv		Ure		Alp		Cat	
		F-value	*p*	F-value	*p*	F-value	*p*	F-value	*p*
LUT	4	25.6	**<0.001**	30.78	**<0.001**	19.53	**<0.001**	2.11	0.098
SD	1	34.83	**<0.001**	56.65	**<0.001**	60.42	**<0.001**	14.84	**<0.001**
LUT × SD	4	7.98	**<0.001**	35.77	**<0.001**	8.81	**<0.001**	3.90	**<0.001**

Table S4. Results of two-way ANOVA for the effects of land use types (LUT) and soil depths (SD) on Shannon and Chao1, F: F-value.

Treatments	df	Shannon		Chao 1	
		F-value	*p*	F-value	*p*
LUT	4	3.4	**0.018**	3.04	**0.028**
SD	1	9.16	**0.004**	5.05	**0.030**
LUT × SD	4	1.07	0.383	1.22	**0.032**
